# Light-Driven Biosynthesis of *myo*-Inositol Directly From CO_2_ in *Synechocystis* sp. PCC 6803

**DOI:** 10.3389/fmicb.2020.566117

**Published:** 2020-09-29

**Authors:** Xiaoshuai Wang, Lei Chen, Jing Liu, Tao Sun, Weiwen Zhang

**Affiliations:** ^1^Laboratory of Synthetic Microbiology, School of Chemical Engineering and Technology, Tianjin University, Tianjin, China; ^2^Frontier Science Center for Synthetic Biology and Key Laboratory of Systems Bioengineering, Ministry of Education of China, Tianjin, China; ^3^Collaborative Innovation Center of Chemical Science and Engineering, Tianjin, China; ^4^School of Life Sciences, Tianjin University, Tianjin, China; ^5^Center for Biosafety Research and Strategy, Tianjin University, Tianjin, China

**Keywords:** *myo*-inositol, cyanobacteria, photosynthetic cell factory, small RNA tools, synthetic biology

## Abstract

*myo*-inositol (MI) is an essential growth factor, nutritional source, and important precursor for many derivatives like D-*chiro*-inositol. In this study, attempts were made to achieve the “green biosynthesis” of MI in a model photosynthetic cyanobacterium *Synechocystis* sp. PCC 6803. First, several genes encoding *myo*-inositol-1-phosphate synthases and *myo*-inositol-1-monophosphatase, catalyzing the first or the second step of MI synthesis, were introduced, respectively, into *Synechocystis*. The results showed that the engineered strain carrying *myo*-inositol-1-phosphate synthase gene from *Saccharomyces cerevisiae* was able to produce MI at 0.97 mg L^–1^. Second, the combined overexpression of genes related to the two catalyzing processes increased the production up to 1.42 mg L^–1^. Third, to re-direct more cellular carbon flux into MI synthesis, an inducible small RNA regulatory tool, based on MicC-Hfq, was utilized to control the competing pathways of MI biosynthesis, resulting in MI production of ∼7.93 mg L^–1^. Finally, by optimizing the cultivation condition via supplying bicarbonate to enhance carbon fixation, a final MI production up to 12.72 mg L^–1^ was achieved, representing a ∼12-fold increase compared with the initial MI-producing strain. This study provides a light-driven green synthetic strategy for MI directly from CO_2_ in cyanobacterial chassis and represents a renewable alternative that may deserve further optimization in the future.

## Introduction

Inositol, known as cyclohexanehexol, is a vital growth factor previously identified in bacteria, fungi, higher plants, and animals. It has nine isomers (i.e., *myo*-, *cis*-, *epi*-, *allo*-, *muco*-, *neo*-, L-*chiro*-, D-*chiro*-, and *scyllo*-), and five of them have been found in nature, namely, D-*chiro*-inositol, L-*chiro*-inositol, *myo*-inositol, *neo*-inositol, and *scyllo*-inositol (Thomas et al., 2016). Among them, *myo*-inositol (*cis*-1, 2, 3, 5-*trans*-4, 6-cyclohexanehexol, hereafter MI) and its derivatives are the most abundant in nature and have attracted significant attention in recent years due to their wide applications in functional food and pharmaceutical industry (You et al., 2017). For example, MI was reported to be effective in restoring spontaneous ovarian activity, consequently improving the fertility of most patients with polycystic ovary syndrome (Regidor et al., 2018; Januszewski et al., 2019). In addition, MI serves as a precursor for many valuable chemicals, further generating numerous important chemicals participating in maintaining homeostasis, such as inositol-1, 4, 5-trisphosphate (IP3) that functions as a Ca^2+^-mobilizing second messenger in regulating many cellular processes (Berridge, 2009). Moreover, MI can also be converted to *scyllo*- and D-*chiro*–inositol, both of which have potential roles in the medicine industry in curing Alzheimer’s disease and hyperglycemia (Ma et al., 2012; Cheng et al., 2019). It is thus valuable to develop cost-efficient strategies for MI production.

Several strategies have been so far reported for MI synthesis. Among all chemical approaches, it is difficult to operate and is less environmentally friendly due to its harsh chemical conditions, such as low pH, high temperature, and high pressure. Recently, the microbial production of MI through synthetic biology has attracted increasing attention (Fujisawa et al., 2017; You et al., 2017; Lu et al., 2018). For example, Lu et al. (2018) recently reported a novel pathway to produce MI from glucose through a trienzymatic cascade system *in vitro*, achieving a productivity of 45.2 mM within 24 h. By dynamically modulating the key enzyme phosphofructokinase-I (Pfk-I) in *Escherichia coli*, recently a level of MI production at 1.31 g L^–1^ was achieved (Brockman and Prather, 2015). In addition, Tanaka et al. (2013) constructed a pathway starting from MI to *scyllo*-inositol in *Bacillus subtilis*, resulting in *scyllo*-inositol productivity of 10 g L^–1^ after 48 h. More recently, by introducing *Mycobacterium tuberculosis ino1* gene encoding *myo*-inositol-1-phosphate synthase and overexpressing intrinsic inositol monophosphatase, *YktC*, as well as an artificial pathway converting *myo*-inositol to *scyllo*-inositol in *Bacillus subtilis*, Michon et al. (2020) achieved a production of 2 g L^–1^ *scyllo*-inositol using 20 g L^–1^ glucose. Nevertheless, even with all the exciting progresses, a new, renewable, and cost-efficient alternative for MI production remains to be developed.

Due to the ability of utilizing sunlight and CO_2_ as sole energy and carbon sources, respectively, cyanobacteria are considered as promising green chassis for producing chemicals. Up to now, several dozens of biofuels and chemicals have been successfully synthesized directly from CO_2_ in cyanobacteria, such as ethylene, ethanol, fatty acids, D-lactic acid, 3-hydroxypropionic acid, etc. (Gao et al., 2016). As a model cyanobacterium, *Synechocystis* sp. PCC 6803 (hereafter *Synechocystis*) has the advantages of a simple genetic background and feasible genetic tools for metabolic engineering and synthetic biology (Sun et al., 2018a). Given that cyanobacteria could directly use CO_2_ to produce chemicals driven by sunlight, attempts were made in this study to construct green synthesis strategy for MI in *Synechocystis* chassis.

**FIGURE 1:**
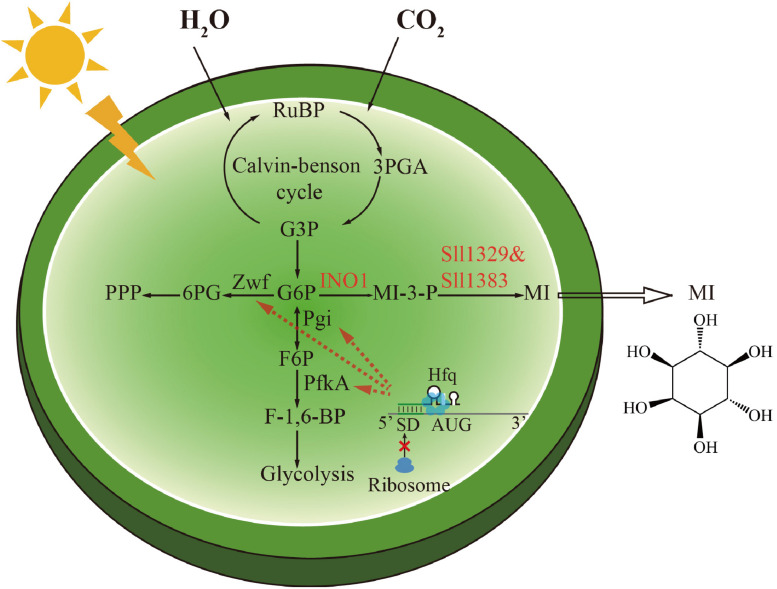
Scheme of the biosynthetic pathway of *myo*-inositol and the metabolic regulation strategy in *Synechocystis*. The genetic modifications made in this study were highlighted. The conversion of glucose-6-phosphate (G-6-P) to *myo*-inositol (MI) was catalyzed by inositol-1-phosphate synthase (INO1) and myo-inositol-1-monophosphatase (Sll1329 and Sll1383). The phosphoglucose isomerase (Pgi), phosphofructokinase (PfkA), and glucose-6-phosphate dehydrogenase (Zwf) were downregulated by a small RNA tool to direct G-6-P to MI biosynthesis.

In this study (Figure 1), to achieve the green synthesis of MI, we first constructed an exogenous metabolic route to convert glucose-6-phosphate to MI in *Synechocystis* by, respectively, introducing *myo*-inositol-1-phosphate synthase from *Saccharomyces cerevisiae* or *Corynebacterium glutamicum* as well as the native genes (*sll1329* and *sll1383*) encoding myo-inositol-1-monophosphatase. The results showed that the engineered *Synechocystis* carrying *INO1* (*myo*-inositol-1-phosphate synthases from *S. cerevisiae*) performed the best, with MI production of 0.97 mg L^–1^. Second, the combined overexpression of *INO1*, *sll1329*, and *sll1383* further improved MI production. Third, to drive more carbon flux into MI synthesis, endogenous gene *zwf* (encoding glucose-6-phosphate dehydrogenase), *pgi* (encoding glucose-6-phosphate isomerase), and *pfkA* (encoding phosphofructokinase) were knocked down, respectively, and combined, using a theophylline-inducible small RNA (sRNA) regulatory tool based on MicC-Hfq, leading to MI production of up to 7.93 mg L^–1^. Finally, by supplying bicarbonate to enhance carbon fixation, a final MI production up to 12.72 mg L^–1^ was achieved, representing a ∼12-fold increase compared with the initial MI-producing strain.

## Materials and Methods

### Chemicals and Reagents

MI standard was purchased from Rhawn Chemical Technology Co., Ltd. (Shanghai, China). The other chemicals used in this study were purchased from Sigma-Aldrich (MO, United States). T4 Polynucleotide Kinase, T4 DNA ligase, and all restriction enzymes were purchased from Thermo Fisher Scientific (MA, United States). Phanta Super-Fidelity DNA Polymerase, ChamQ SYBR qPCR Master Mix, and HiScript Q RT SuperMix for qPCR were obtained from Vazyme Biotech Co., Ltd. (Nanjing, China). The Plasmid Mini Kit I and Cycle Pure Kit used were purchased from Omega Bio-Tek (GA, United States). Synthesis of DNA oligonucleotide primers and Sanger sequencing were provided by Genewiz (Suzhou, China).

### Culture Conditions

The wild-type (WT) and all engineered strains of *Synechocystis* were grown at 30°C in BG-11 liquid medium or on solid BG-11 agar plate at a light intensity of ∼50 μmol photons m^–2^ s^–1^ in an incubator (SPX-250B-G, Boxun, Shanghai, China) or illuminating shaking incubator (HNY-211B, Honour, Tianjin, China) at 130 rpm, respectively. Appropriate antibiotic(s) was added into the BG-11 growth medium as required (i.e., 20 μg ml^–1^ chloramphenicol or 20 μg ml^–1^ spectinomycin). The growth of the cells was monitored by measuring their optical density at 730 nm (OD_730_) with a UV-1750 spectrophotometer (Shimadzu, Kyoto, Japan). *E. coli* Trans 5α was used as a host for constructing all recombinant plasmids, which were grown on Luria–Bertani solid agar plates or in a medium with appropriate antibiotic(s) to maintain the plasmids (i.e., 50 μg ml^–1^ chloramphenicol or 50 μg ml^–1^ spectinomycin) at 37°C in an incubator or a shaking incubator (HNY-100B, Honour, Tianjin, China) at 200 rpm, respectively.

### Strain and Plasmid Construction

*E. coli* Trans 5α was used as a host for plasmid construction and amplification. In this study, two suicide plasmids, p3031 and p0168, that could replicate in *E. coli* and integrate into the genome of *Synechocystis* (between *slr2030* and *slr2031* for p3031 or within *slr0168* for p0168, respectively) via homologous recombination were utilized to express the related genes. The *INO1* and *cgl2996* genes were amplified using *S. cerevisiae* and *C. glutamicum* genomic DNA as templates, respectively. Then *INO1* and *cgl2996* were, respectively, ligated into pCP3031 (Supplementary Figure S1), resulting in plasmids p3031I and p3031C, respectively. After being confirmed by DNA sequencing, these two genes were, respectively, introduced into WT, generating the strains WT-INO1 and WT-cgl. The *sll1329* and *sll1383* genes were amplified using *Synechocystis* genomic DNA as template and fused into one fragment linked by a ribosome binding site (RBS) via overlapping PCR. The fused fragment was then ligated into pCP3031, resulting in plasmid p3031SS. The p3031SS was introduced into WT, generating the strain WT-SS. In addition, the fragments of *sll1329* and *sll1383* were further fused with a strong promoter, Pcpc560, and inserted after the cassette of *INO1* on p3031I for transformation, generating the plasmid p3031S and the *Synechocystis* strain WT-INO1SS, respectively. The construction of sRNA-expressing plasmids was conducted as reported previously (Sun et al., 2018b). First, a light-induced promoter PpsbA2M (Ppsba2 without RBS) was utilized to express the sRNA scaffold *micC*, while a theophylline-induced riboswitch was used to control the expression of *hfq*, respectively (Supplementary Figure S2). Second, the synthetic 24-bp sRNA sequence (*aszwf*) targeting the translational starting site of the *zwf* was added into the location between PpsbA2M and MicC, leading to p0168Z, with a PpsbA2M-*aszwf-micC*-TrbcL-Ptrc-riboswitch-*hfq*-TrbcL cassette. Similarly, the plasmids with sRNA-expressing cassette targeting the *pgi* and the *pfkA* (*aspgi* and *aspfkA*) were constructed independently, leading to p0168P and p0168PF, respectively. The p0168Z, p0168P, and p0168PF plasmids were, respectively, transferred into *Synechocystis* WT-INO1SS through natural transformation, generating the strains WT-INO1SS-ASZWF, WT-INO1SS-ASPGI, and WT-INO1SS-ASPFKA, respectively. Finally, two expressing cassettes, PpsbA2M-*aspgi-micC*-TrbcL and PpsbA2M-*aspfkA-micC*-TrbcL, were ligated into p0168Z, generating p0168ZP or p0168ZF, respectively targeting two genes (i.e., targeting *zwf* and *pgi* or *zwf* and *pfkA*). They were then introduced into WT-INO1SS to generate the strains WT-INO1SSZP and WT-INO1SSZF, respectively. All the strains and the plasmids used and constructed in this study are listed in Table 1.

**TABLE 1:** Strains used in this study.

Strains	Genotype	References
*Escherichia coli* Trans5α	F^–^, φ80d *lac*ZΔM15, Δ(*lac*ZYA-*arg*F) U169, *end*A1, *rec*A1, *hsd*R17 (rk^–^, mk^+^), *sup*E44λ, *thi*-1, *gyr*A96, *rel*A1, *pho*A	Stratagene
**Cyanobacteria strains**		
*Synechocystis* sp. PCC 6803	WT	ATCC 27184
WT-INO1	*slr2030*_*slr2031*:Pcpc560-*INO1*-TrbcL; Spe^R^ in WT	This study
WT-cgl	*slr2030*_*slr2031*:Pcpc560-*cgl2996*-TrbcL; Spe^R^ in WT	This study
WT-INO1SS	*slr2030*_*slr2031*:Pcpc560-*INO1*-TrbcL-Pcpc560- *sll1329*-rbs-*sll1383*-TrbcL; Spe ^R^ in WT	This study
WT-SS	*slr2030*_*slr2031*:Pcpc560-*sll1329*-rbs- *sll1383*-TrbcL; Spe ^R^ in WT	This study
WT-INO1SS-ASZWF	*slr0168*:PpsbA2M-*aszwf-micC*-TrbcL-Ptrc-riboswitch-*hfq*-TrbcL; Cm^R^ in WT-INO1SS	This study
WT-INO1SS-ASPGI	*slr0168*:PpsbA2M-*aspgi-micC*-TrbcL-Ptrc-riboswitch-*hfq*-TrbcL; Cm^R^ in WT-INO1SS	This study
WT-INO1SS-ASPFKA	*slr0168*:PpsbA2M-*aspfkA-micC*-TrbcL-Ptrc-riboswitch-*hfq*-TrbcL; Cm^R^ in WT-INO1SS	This study
WT-INO1SSZP	*slr0168*:PpsbA2M-*aszwf-micC*-TrbcL- PpsbA2M-*aspgi-micC*-TrbcL-Ptrc-riboswitch-*hfq*-TrbcL; Cm^R^ in WT-INO1SS	This study
WT-INO1SSZF	*slr0168*:PpsbA2M-*aszwf-micC*-TrbcL- PpsbA2M-*aspfkA-micC*-TrbcL Ptrc-riboswitch-*hfq*-TrbcL; Cm^R^ in WT-INO1SS	This study

### Transformation of *Synechocystis*

Natural transformation of *Synechocystis* was performed according to the method published previously. Briefly, when *Synechocystis* grew to exponential phase (OD_730_≈ 0.5), cells were collected by centrifugation (3,000 × *g*, 13 min, 4°C) and washed with fresh BG-11 medium. The cells were then resuspended in fresh BG-11, and ∼10 μg of corresponding plasmid DNA was added to the suspension. The cell and plasmid mixture was incubated at 30°C for at least 5 h under luminous intensity of ∼50 μmol photons m^–2^ s^–1^, followed by spreading onto BG-11 agar plates with appropriate antibiotic(s) (e.g., 20 μg ml^–1^ chloramphenicol and/or 20 μg ml^–1^ spectinomycin). After incubation of ∼2 weeks, colonies were observed. After validation by colony PCR and sequencing, positive colonies would be transferred to liquid BG11 medium for growth and further examination.

### MI Quantification

For *Synechocystis* samples, 1 ml of fresh cultures of *Synechocystis* was collected on the third day by centrifugation at 12,000 × *g* for 5 min at room temperature (Eppendorf 5430R, Hamburg, Germany). The MI content in the sample pellets and the supernatant were, respectively, measured after performing pre-column derivatization according to the two-stage technique described previously (Roessner et al., 2001). Meanwhile, the stock solution of MI was prepared in ddH_2_O at a final concentration of 1 g L^–1^. The MI standard curve was plotted using different concentrations of MI solution (Supplementary Figure S4). MI levels were quantified on a gas chromatography–mass spectrometry system—GC 7890 coupled to MSD 5975 (Agilent Technologies, Inc., Santa Clara, CA) equipped with a HP-5MS capillary column (30 m × 250 mm id).

### Theophylline Treatment

The stock solution of theophylline was prepared by dissolving theophylline (Aladdin; Shanghai; China) in BG-11 medium at a final concentration of 10 mM. For theophylline-induced assays, all *Synechocystis* samples were collected by centrifugation at 3,000 × *g* and 4°C for 12 min and then re-suspended using fresh BG-11 medium with stock solution of theophylline at a final concentration of 2 mM (Sun et al., 2018b).

### Quantitative Real-Time PCR Analysis

*Synechocystis* samples were collected at 24 h after 2 mM theophylline induction. ∼5 ml of samples (OD_730_ = 1.0) was collected by centrifugation at 3,000 × *g* and 4°C for 12 min. The supernatant was removed, and the cell pellet was used for RNA extraction. Total RNA extraction was achieved through a Direct-zol^TM^ RNA MiniPrep Kit (Zymo, CA, United States), and cDNAs were synthesized using HiScript Q RT SuperMix for qPCR (Vazyme Biotech Co., Ltd., Nanjing, China). The 10-μl qRT-PCR reaction included 5 μl ChamQ SYBR qPCR Master Mix (Vazyme Biotech Co., Ltd., Nanjing, China), 3 μl ddH_2_O, 1 μl diluted template cDNA, and 1 μl of each PCR primer (0.5 μl forward primer and 0.5 μl reverse primer). The reaction was conducted in the StepOne^TM^ Real-Time PCR System (Applied Biosystems, CA, United States). 16S rRNA was selected as the reference gene, and the primers of the specific genes used are listed in Supplementary Table S1. Data analysis was carried out by using 2^−ΔΔ^^CT^ method as reported previously (Livak and Schmittgen, 2001).

## Results

### Construction of MI-Producing *Synechocystis*

The bioconversion from glucose to MI involves three steps which are catalyzed by three sequentially acting enzymes (Fujisawa et al., 2017; You et al., 2017; Lu et al., 2018). First, with the aid of hexokinase, glucose was phosphorylated to glucose-6-phosphate (G6P). Second, G6P was converted into *myo*-inositol 3-phosphate (I3P), catalyzed by inositol-1-phosphate synthase (IPS), which was responsible for the committed step of inositol synthesis. Third, I3P was dephosphorylated to generate MI by inositol-1-monophosphatase. Previously, native genes potentially related to MI synthesis have been identified in *Synechocystis* (Chatterjee et al., 2004, 2006; Patra et al., 2007). In this study, to detect the production of MI in the *Synechocystis* WT strain, we measured both intracellular and extracellular MI, and the results showed that no detectable MI could be observed even after 7 days of cultivation, demonstrating that the MI produced natively was below the detection limit. Previously, studies showed that the *INO1* gene encoding inositol-1-phosphate synthase from *S. cerevisiae* could perform well in *E. coli* (Gupta et al., 2017). In addition, an IPS gene of the same function, *cgl2996*, was also identified in *C. glutamicum*. Therefore, *INO1* from *S. cerevisiae* and *cgl2996* from *C. glutamicum* were, respectively, introduced and evaluated in *Synechocystis*, generating the strains WT-INO1 and WT-cgl (Table 1).

**FIGURE 2:**
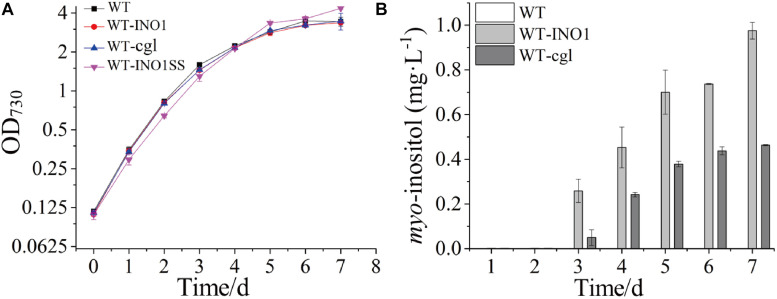
Growth curves and *myo*-inositol quantitation in wild-type (WT) and engineered *Synechocystis* strains. The error bar represents the standard deviation of three biological replicates for each sample. **(A)** Growth curves of WT, WT-INO1, WT-cgl, and WT-INO1SS. **(B)** *myo*-inositol quantitation in WT and WT-cgl.

The growth comparison between WT, WT-INO1, and WT-cgl suggested that overexpression of neither *INOI* nor *cgl2996* affected the growth of the engineered strains (Figure 2A). The potential production of MI was measured in both WT-INO1 and WT-cgl strains. As shown in Figure 2B, no MI was detected by GC-MS in the first 2 days as they were probably below the detection limit; however, MI could be observed in both intracellular and extracellular samples from the 3rd day (Supplementary Figure S3) and accumulated steadily until the 7th day (stationary phase) (Figure 2B). Finally, after cultivation for 7 days, the production of MI reached ∼975.5 μg L^–1^ in WT-INO1, while it only reached ∼463.8 μg L^–1^ in WT-cgl, respectively. These results demonstrated that the introduction of exogenous IPS could enable the MI biosynthesis in *Synechocystis*. In addition, the IPS from *S. cerevisiae* seemed to function better for MI biosynthesis than that from *C. glutamicum* in *Synechocystis*.

### Enhancement of MI Production by Overexpressing Key Genes

**FIGURE 3:**
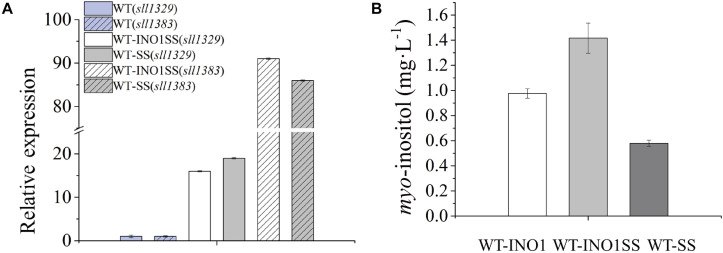
qRT-PCR assays and *myo*-inositol quantitation in the engineered *Synechocystis* strain WT-INO1SS. The error bar represents the standard deviation of three technical replicates for each sample. **(A)** Relative transcriptional level of *sll1383* and *sll1329* in WT and WT-INO1SS, respectively. **(B)** *myo*-inositol quantitation in WT-INO1, WT-SS, and WT-INO1SS.

*myo*-inositol-1-monophosphatase (IMP) is another crucial enzyme for MI synthesis that catalyzes the production of *myo*-inositol from *myo*-inositol 3-phosphate. This enzyme is putatively encoded by *sll1329* or *sll1383* in *Synechocystis* according to a previous study (Patra et al., 2007) and the KEGG pathway annotation^1^. In order to further improve the MI production, we simultaneously overexpressed the genes *sll1329* and *sll1383* (isozyme genes, both encoding IMP) using a strong promoter Pcpc560 in WT-INO1, resulting in the strain WT-INO1SS (Table 1). As a control, strain WT-SS with only overexpressed *sll1329* and *sll1383* was also constructed (Table 1). To evaluate their expression, the transcriptional levels of *sll1329* and *sll1383* in WT-INO1SS or WT-SS were quantified via qRT-PCR. As illustrated in Figure 3A, the transcriptional levels of *sll1329* and *sll1383* were, respectively, increased by more than 15- and 80-folds in both WT-INO1SS and WT-SS compared to that of WT, suggesting a successful overexpression. The growth of WT and WT-INO1SS was comparatively investigated, and the results showed that the overexpression of *sll1329* and *sll1383* caused no visible growth inhibition (Figure 2A). Moreover, the production of MI reached 1.42 mg L^–1^ in WT-INO1SS after cultivation for 7 days, achieving ∼45.6% increase compared to that in WT-INO1 (Figure 3B). In the control WT-SS strain where only *sll1329* and *sll1383* were expressed, MI analysis showed that it can produce MI production at 579.6 μg L^–1^, confirming the existence of a native *INO1* gene encoding inositol-1-phosphate synthase in *Synechocystis* (Chatterjee et al., 2004, 2006; Patra et al., 2007), although its catalytical activity might be well lower than INO1 from *S. cerevisiae* and Cgl2996 from *C. glutamicum*. The results supported the report that the overexpression of *sll1329* and *sll1383* could improve the production of MI.

### Re-direction of Carbon Flux Toward MI Production

Glycolysis and pentose phosphate pathway are the main carbon source competing pathways for MI synthesis (Figure 1; Hansen et al., 1999). However, total blocking of these essential pathways would cause severe growth inhibition or even a lethal phenotype. In order to drive more carbon flux from the competing pathways to MI biosynthesis, a sRNA tool MicC–Hfq, developed previously in *Synechocystis*, that allows “gene knock-down” was adopted (Sun et al., 2018b). In detail, the Hfq–MicC tool is composed of a chaperone protein Hfq and a well-studied sRNA scaffold named MicC from *E. coli*. With a designed target-binding region fused into the MicC scaffold, the fragment could regulate the expression of target genes effectively via altering their translation with the aid of the Hfq chaperone.

**FIGURE 4:**
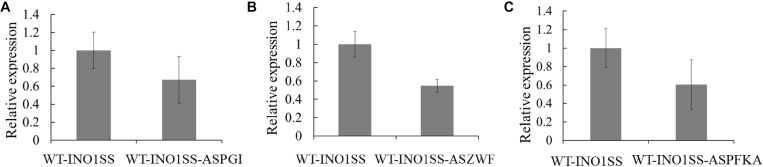
qRT-PCR assays in the engineered *Synechocystis* strains WT-INO1SS-ASPGI, WT-INO1SS-ASZWF, and WT-INO1SS-ASPFKA after providing the supplement of 2 mM theophylline. The error bar represents the standard deviation of three technical replicates for each sample. **(A)** Relative transcriptional level of *pgi* in WT-INO1SS and WT-INO1SS-ASPGI. **(B)** Relative transcriptional level of *zwf* in WT-INO1SS and WT-INO1SS-ASZWF. **(C)** Relative transcriptional level of *pfkA* in WT-INO1SS and WT-INO1SS-ASPFKA.

**FIGURE 5:**
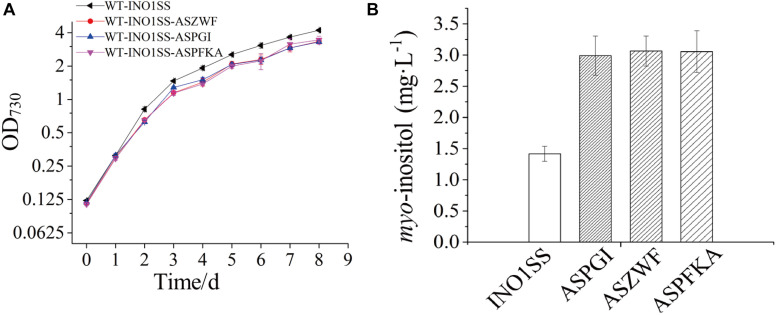
Growth curves and *myo*-inositol quantitation in the engineered *Synechocystis* strains WT-INO1SS-ASPGI, WT-INO1SS-ASZWF, and WT-INO1SS-ASPFKA. The error bar represents the standard deviation of three biological replicates for each sample. INO1SS, strain WT-INO1SS; ASPGI, strain WT-INO1SS-ASPGI; ASZWF, strain WT-INO1SS-ASZWF; ASPFKA, strain WT-INO1SS-ASPFKA. **(A)** Growth curves of WT-INO1SS, WT-INO1SS-ASPGI, WT-INO1SS-ASZWF, and WT-INO1SS-ASPFKA. **(B)** *myo*-inositol quantitation in WT-INO1SS, WT-INO1SS-ASPGI, WT-INO1SS-ASZWF, and WT-INO1SS-ASPFKA after 8 days of cultivation.

Glucose-6-phosphate (G6P) is not only a metabolic branch point but also a substrate for inositol synthesis in cells, as it could be routed into native cellular metabolism through both glycolysis and the oxidative pentose phosphate pathway, as well as into the heterologous biosynthetic pathway of MI production. Thus, *pgi* (encoding phosphoglucose isomerase), *pfkA* (encoding phosphofructokinase), and *zwf* (encoding glucose-6-phosphate dehydrogenase) were chosen as the target genes, and the related sRNA expressing systems were constructed. The synthetic sRNA sequences targeting the translational starting site of the *zwf*, *pgi*, or *pfkA* were fused, respectively, into the MicC scaffold and driven by PpsbA2M (PpsbA2 without RBS), while the expression of *hfq* was controlled by the Ptrc containing a theophylline-induced riboswitch. In addition, the related strains WT-INO1SS-ASPGI, WT-INO1SS-ASPFKA, and WT-INO1SS-ASZWF were, respectively, achieved via introducing the MicC–Hfq-expressing cassettes (Table 1). After induction with 2 mM theophylline, qRT-PCR analysis, growth curves, and *myo*-inositol production determination of the three strains were performed. First, qRT-PCR analysis was performed to validate the knockdown effect of the MicC–Hfq tool on all three target genes. As shown in Figures 4A–C, when compared to that in WT-INO1SS, 33, 45, and 39% down-regulation for *pgi*, *pfkA*, and *zwf* were achieved via the synthetic sRNA in WT-INO1SS-ASPGI, WT-INO1SS-ASPFKA, and WT-INO1SS-ASZWF, respectively. Second, the growth rate of the three strains was comparatively monitored, and only a slight inhibition was observed for WT-INO1SS-ASPGI, WT-INO1SS-ASPFKA, and WT-INO1SS-ASZWF compared to WT-INO1SS (Figure 5A). Third, the production of MI in three strains was determined, and the results showed that MI production was increased to 3.00, 3.06, and 3.06 mg L^–1^ in strains WT-INO1SS-ASPGI, WT-INO1SS-ASZWF, and WT-INO1SS-ASPFKA, respectively (Figure 5B), suggesting that the knock-down of *pgi*, *zwf*, and *pfkA* by the synthetic sRNAs was able to efficiently increase the metabolic flux from glucose-6-phosphate into MI.

**FIGURE 6:**
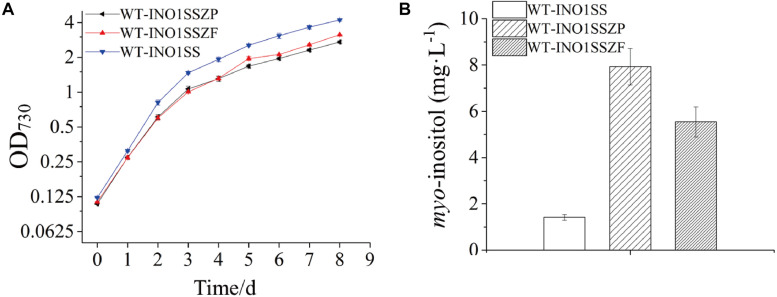
Growth curves and *myo*-inositol quantitation in the engineered *Synechocystis* strains WT-INO1SSZP and WT-INO1SSZF. The error bar represents the standard deviation of three biological replicates for each sample. **(A)** Growth curves of WT-INO1SS, WT-INO1SSZP, and WT-INO1SSZF. **(B)** *myo*-inositol quantitation in WT-INO1SS, WT-INO1SSZP, and WT-INO1SSZF after 8 days of cultivation.

Given that *pgi* and *pfkA* genes are both involved in the same pathway, a combined regulation for *zwf* and *pgi* or *zwf* and *pfkA* was carried out to evaluate whether it can further increase MI production. Accordingly, two strains, WT-INO1SSZP and WT-INO1SSZF, carrying the synthetic sRNAs targeting both *zwf* and *pgi* or both *zwf* and *pfkA*, respectively, were constructed. As illustrated in Supplementary Figure S5, the expression level of *zwf* was decreased by 42 and 41% in WT-INO1SSZP and WT-INO1SSZF; *pfkA* in WT-INO1SSZF and *pgi* in WT-INO1SSZP were decreased as well by 39 and 37% compared to that in WT after induction with 2 mM theophylline. The growth curves and the MI yields of the two strains were then measured after addition of 2 mM theophylline (Figure 6A); however, an obvious retardation in growth was observed for both strains, possibly due to the increased partitioning of carbon sources toward *myo*-inositol, which is consistent with the increased MI production in WT-INO1SSZP and WT-INO1SSZF even with the decreased growth (Figure 6B). The results showed that the MI production reached 7.93 and 5.54 mg L^–1^ in WT-INO1SSZP and WT-INO1SSZF, respectively, indicating that the combined regulation of *zwf* and *pgi* was more effective for increasing MI synthesis. Between the two engineered strains, WT-INO1SSZP grew slower than WT-INO1SSZF (Figure 6A), which may be due to the fact that more glucose-6-phosphate was directed into the MI biosynthesis pathway from glycolysis and pentose phosphate pathway with the aid of sRNA tools in WT-INO1SSZP.

### Cultivation Optimization to Enhance MI Production

**FIGURE 7:**
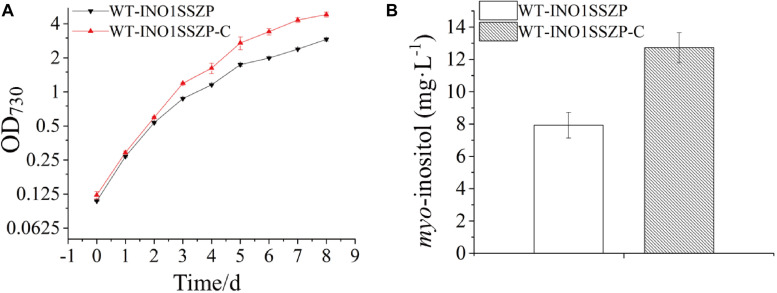
*myo*-inositol quantitation and growth curves in the engineered *Synechocystis* strain WT-INO1SSZP, cultivated with or without NaHCO_3_. The error bars represent the standard deviation of three biological replicates for each sample. WT-INO1SSZP represents cultivation without NaHCO_3_, while WT-INO1SSZP-C represents cultivation with NaHCO_3_. **(A)** Growth curves of WT-INO1SSZP with or without NaHCO_3_. **(B)** *myo*-inositol quantitation in WT-INO1SSZP after cultivation for 8 days with or without NaHCO_3_.

Early studies have demonstrated that NaHCO_3_ supplementation to cyanobacterial culture is an effective strategy for higher biomass and more production of target chemicals (Johnson et al., 2016; Wang et al., 2016). Thus, the effects of increased NaHCO_3_ supply on MI production in the engineered strain were evaluated. Given that the strain WT-INO1SSZP showed the highest MI production capacity, it was chosen as the target for cultivation optimization. After supplementing 0.5 ml 1.0 M NaHCO_3_ every 24 h into the BG-11, the engineered *Synechocystis* strain WT-INO1SSZP showed a faster growth rate compared with their corresponding strains without NaHCO_3_ supplementation (Figure 7A). Meanwhile, the MI production in the strain WT-INO1SSZP was increased to 12.72 mg L^–1^ (Figure 7B), demonstrating that the enhanced carbon supply could significantly increase the MI production.

## Discussion

Early studies have shown the feasibility of directly converting light energy and CO_2_ into green fuels and chemicals in *Synechocystis* (Gao et al., 2016; Wang et al., 2016). In this study, we engineered a photoautotrophic cyanobacterial system for the production of MI directly from CO_2_. Previously, *INO1* and *myo*-inositol 1-phosphate synthase were overexpressed from *S. cerevisiae* in *E. coli* (Brockman and Prather, 2015), successfully achieving a heterologous production of MI. Consistently, we found that the overexpression of *INO1* led to detectable MI biosynthesis in *Synechocystis*. After the overexpression of *sll1329* and *sll1383* (encoding *myo*-inositol-1-monophosphatase), intracellular MI concentration was slightly increased by ∼45.6% in WT-INO1SS than that in WT-INO1. A significant overexpression for *sll1329* and *sll1383* on a transcriptional level was demonstrated via qRT-PCR (Figure 3), suggesting that IMP might not be the limiting step for MI synthesis in *Synechocystis*.

G6P was the direct precursor for MI synthesis; meanwhile, it is a fundamental metabolite to support microbial survival. Though the manipulation of carbon flux toward the G6P pool has previously been demonstrated to be an effective strategy to enhance the production of its derivatives in various microorganisms (Brockman and Prather, 2015; Gupta et al., 2017), deletion of the pentose phosphate pathway (PPP)-related gene *zwf* could totally block the essential pathways and cause severe growth inhibition. Thus, a suitable and efficient genetic tool for gene knockdown is valuable. Previously, the small RNA regulatory tool was demonstrated as feasible and efficient in regulating genes, especially essential genes, such as redirecting the carbon flux to the key precursor malonyl-CoA in *Synechocystis* (Sun et al., 2018b). In this work, the sRNA tool was utilized to decrease the flux to glycolysis and pentose phosphate pathway based on the theophylline-inducible riboswitch. Interestingly, the down-regulation of either PPP (zwf) or glycolysis pathway (*pgi* or *pfk*A) led to an ∼2-fold increase in MI production compared to that of WT-INO1SS, while the combined regulation of the two pathways realized a synergetic effect with ∼5.58-fold increase of MI production. The results demonstrated that control of the competing pathways and driving more carbon into MI biosynthesis were important for MI production. In the future, attempts could be made to target more “carbon-consuming pathways” like glycogen, fatty acids, as well as acetate synthesis to direct more carbon into MI synthesis (Zhou et al., 2014). Meanwhile, in this study, the control for competing pathways was achieved using an inducible riboswitch, which needs additional inducers at specific time points. As artificial quorum sensing systems allowed cell growth at low cell density and induced specific gene expressions at high cell density automatically (Kim et al., 2017; Gu et al., 2020), it may represent a more suitable switch to control the essential pathways and is worth investigating in the future.

Limiting the carbon flux into other pathways was efficient for enhancing MI synthesis, while improving the total carbon fixation could also be important as it provides more carbon precursors. Previously, the overexpression of genes encoding ribulose-1,5-bisphosphate carboxylase/oxygenase or extra bicarbonate transporters were both demonstrated as feasible for enhancing carbon fixation and biomass accumulation in *Synechocystis* (Atsumi et al., 2009; Kamennaya et al., 2015; Liang and Lindblad, 2016). In addition, supplementation of inorganic carbon like CO_2_ or bicarbonate has been considered as a more direct strategy for carbon fixation reinforcement and production improvement (Wang et al., 2013). Consistently, supplementation of bicarbonate for the cultivation of WT-INO1SSZP further reached a ∼1.6-fold increase in MI production. Nevertheless, the final production in *Synechocystis* is still much lower than that in other heterotrophic microorganisms like *B. subtilis* and *E. coli* (Brockman and Prather, 2015; Michon et al., 2020), suggesting that less than enough carbon sources and precursors were fed into the synthetic pathway. In the future, cultivation supplemented with organic carbon sources like glucose, used for heterotrophic organisms or photomixotrophic, could also be a feasible strategy (Vassilev et al., 2017; Qian et al., 2020). In this work, our preliminary results showed that supplementation of 5 mM glucose could significantly improve the growth and MI production of WT-INO1SS-ZP, finally reaching a production at 21.74 mg L^–1^ after 8 days of cultivation (Supplementary Figure S6), which also supported the argument that more carbon sources and precursors are needed to achieve high MI production.

The relatively lower growth rate of *Synechocystis* could also be an important limiting factor for MI production. Previously, a fast-growing cyanobacterium named *Synechococcus elongatus* UTEX 2973 (Yu et al., 2015; Lin et al., 2020) was isolated, whose shortest doubling time can reach 1.5 h at 41°C under continuous 1,500 μmol photons m^–2^ s^–1^ white light with 5% CO_2_, close to that of *S. cerevisiae* (1.67 h). More importantly, the potential of *S. elongatus* UTEX 2973 for products like sucrose was found to be 6- to 26-fold compared with those in traditional cyanobacterial chassis like *Synechocystis*, *Anabaena* sp. PCC 7120, and *S. elongatus* PCC 7942 (Song et al., 2016), suggesting its application potential for chemical synthesis. Similarly, other fast-growing cyanobacteria like *S. elongatus* PCC 11802 and *Synechococcus* sp. PCC 11901 were identified recently, offering more candidates as chassis for MI production in the future (Jaiswal et al., 2020; Wlodarczyk et al., 2020).

## Conclusion

In this study, we engineered the model cyanobacterium *Synechocystis* for the sustainable production of MI. With the expression of IPS and IMP genes, simultaneous knockdown of three genes related to competing pathways, and cultivation optimization, photosynthetic production of MI directly from CO_2_ was achieved, with a production of up to 12.72 mg L^–1^ after cultivation for 8 days, which represents an increase of ∼12 times compared with initial MI-producing WT-INO1. The study presented here demonstrated the feasibility of converting CO_2_ directly into MI in cyanobacterial chassis.

## Data Availability Statement

All datasets generated for this study are included in the article/Supplementary Material.

## Author Contributions

XW conducted the experiments, analyzed the data, and wrote the manuscript. LC and TS designed the research and revised the manuscript. JL helped with some of the experiments. WZ designed the research and revised the manuscript. All authors contributed to the article and approved the submitted version.

## Conflict of Interest

The authors declare that the research was conducted in the absence of any commercial or financial relationships that could be construed as a potential conflict of interest.

## Footnotes

1 www.genome.jp/kegg-bin/show_pathway?syn00562
