# Supplementary material for: Light-Driven Biosynthesis of myo-Inositol Directly From CO2 in Synechocystis sp. PCC 6803
Source: Front Microbiol. 2020 Sep 29;11:566117. doi: 10.3389/fmicb.2020.566117 (PMC7550737; doi:10.3389/fmicb.2020.566117)
Supplement: Supplementary file 1 [file Table_1.DOCX]

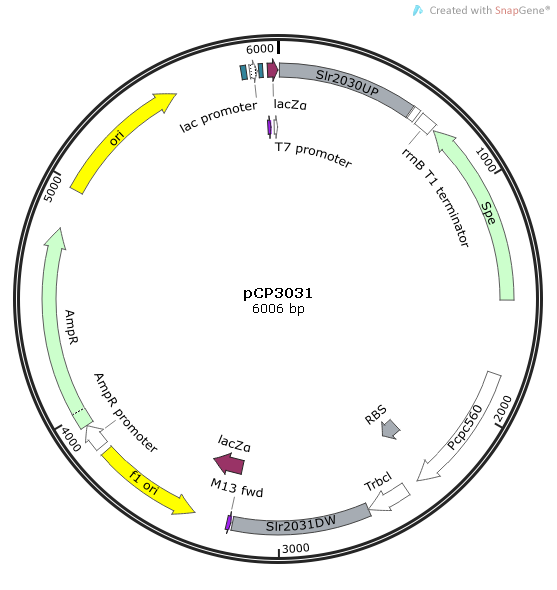


**Figure S1 Schematic of the vector pCP3031.**

**
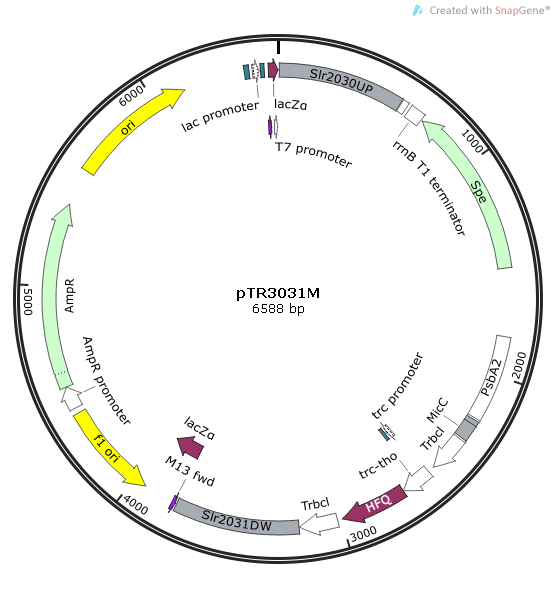
**

**Figure S2 Schematic of the vector pTR3031M.**

**
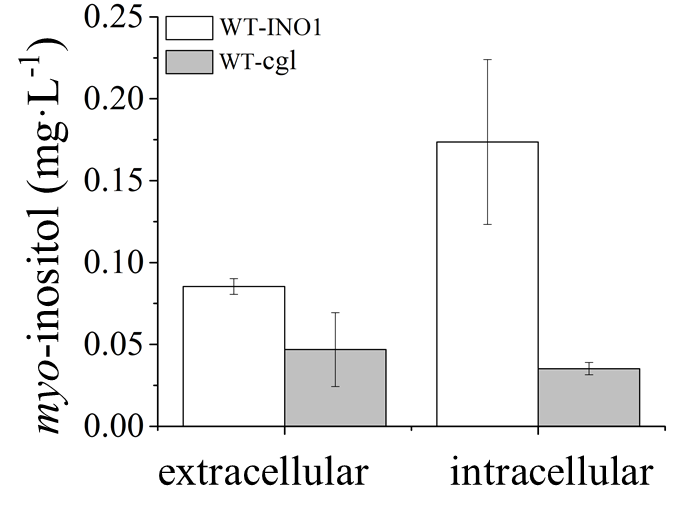
**

**Figure S3 Quantitation of *myo*-inositol in extracellular and intracellular of WT and WT-cgl, respectively.**

**
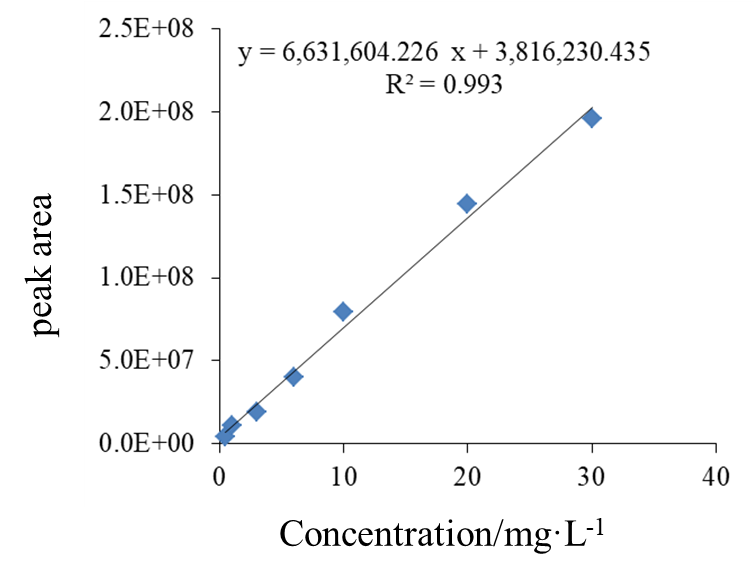
**

**Figure S4 Standard curve of different concentrations of *myo*-inositol.**

**
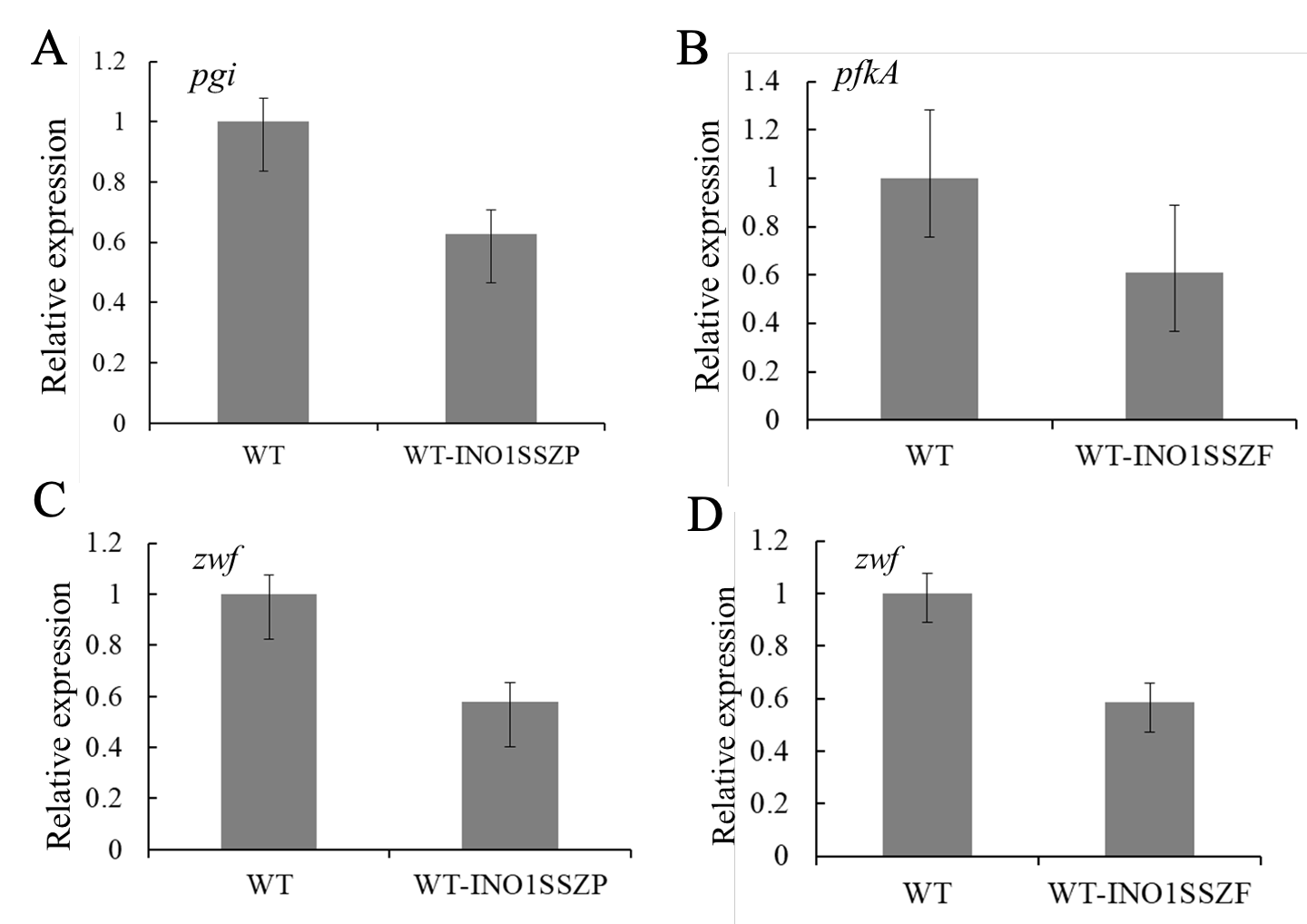
**

**Figure S5 Relevant qRT-PCR assays conducted in *Synechocystis*.** (A) Relative transcriptional level of *pgi* in WT and WT-INO1SSZP, respectively. (B) Relative transcriptional level of *pfkA* in WT and WT-INO1SSZF, respectively. (C) Relative transcriptional level of *zwf* in WT and WT-INO1SSZP, respectively. (D) Relative transcriptional level of *zwf* in WT and WT-INO1SSZF, respectively.

**
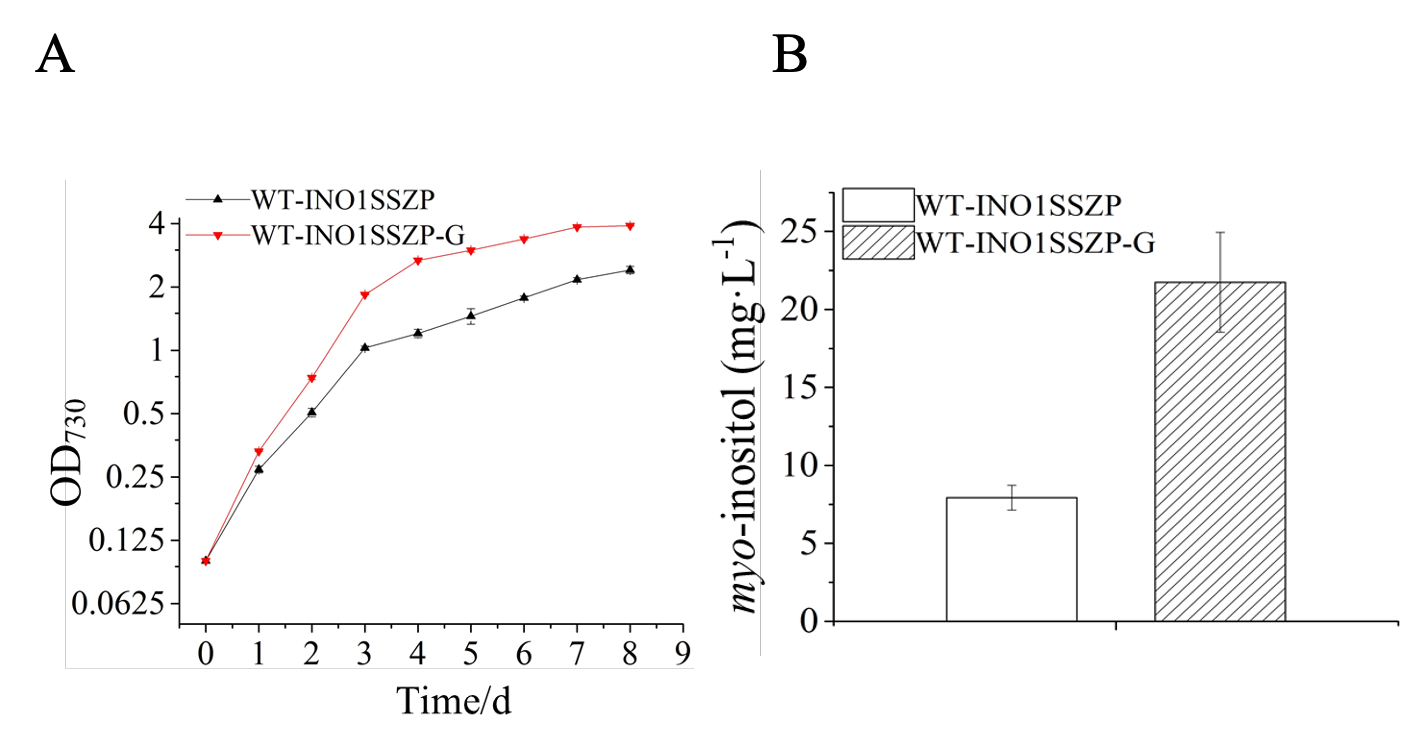
**

**FIGURE S6** ***Myo*-inositol quantitation and growth curves in the engineered *Synechocystis* strains WT-INO1SSZP, cultivated with or without glucose.** The error bar represents the standard deviation of three biological replicates for each sample. “WT-INO1SSZP” means without glucose, WT-INO1SSZP-G” means with glucose. (A) Growth curves of WT-INO1SSZP with or without glucose. (B) *Myo*-inositol quantitation in WT-INO1SSZP on 8 d with or without glucose.
